# Memory-related subjective cognitive symptoms in the adult population: prevalence and associated factors – results of the LIFE-Adult-Study

**DOI:** 10.1186/s40359-018-0236-1

**Published:** 2018-05-21

**Authors:** Tobias Luck, Susanne Roehr, Francisca S. Rodriguez, Matthias L. Schroeter, A. Veronica Witte, Andreas Hinz, Anja Mehnert, Christoph Engel, Markus Loeffler, Joachim Thiery, Arno Villringer, Steffi G. Riedel-Heller

**Affiliations:** 1 Department of Economic and Social Sciences & Institute of Social Medicine, Rehabilitation Sciences and Healthcare Research (ISRV), University of Applied Sciences Nordhausen, Weinberghof 4, 99734 Nordhausen, Germany; 2 Institute of Social Medicine, Occupational Health and Public Health (ISAP), University of Leipzig, Leipzig, Germany; 3 LIFE – Leipzig Research Center for Civilization Diseases, University of Leipzig, Leipzig, Germany; 4 Center for Cognitive Science, University of Kaiserslautern, Kaiserslautern, Germany; 5 Max Planck Institute for Human Cognitive and Brain Sciences, Leipzig, Germany; 6 Clinic for Cognitive Neurology, University Hospital Leipzig, Leipzig, Germany; 7 Collaborative Research Centre 1052 “Obesity Mechanisms”, University of Leipzig, Leipzig, Germany; 8 Department of Medical Psychology and Medical Sociology, University of Leipzig, Leipzig, Germany; 9 Institute for Medical Informatics, Statistics and Epidemiology (IMISE), University of Leipzig, Leipzig, Germany; 10 Institute of Laboratory Medicine, Clinical Chemistry and Molecular Diagnostics (ILM), University Hospital Leipzig, Leipzig, Germany

**Keywords:** Subjective cognitive symptoms, Prevalence, Subjective cognitive decline, Memory, Cognitive performance, Cognitive function, Depression, Comorbidity, Risk factor

## Abstract

**Background:**

Subjectively perceived memory problems (memory-related Subjective Cognitive Symptoms/SCS) can be an indicator of a pre-prodromal or prodromal stage of a neurodegenerative disease such as Alzheimer’s disease. We therefore sought to provide detailed empirical information on memory-related SCS in the dementia-free adult population including information on prevalence rates, associated factors and others.

**Methods:**

We studied 8834 participants (40–79 years) of the population-based LIFE-Adult-Study. Weighted prevalence rates with confidence intervals (95%-CI) were calculated. Associations of memory-related SCS with participants’ socio-demographic characteristics, physical and mental comorbidity, and cognitive performance (Verbal Fluency Test Animals, Trail-Making-Test, CERAD Wordlist tests) were analyzed.

**Results:**

Prevalence of total memory-related SCS was 53.0% (95%-CI = 51.9–54.0): 26.0% (95%-CI = 25.1–27.0) of the population had a subtype without related concerns, 23.6% (95%-CI = 22.7–24.5) a subtype with some related concerns, and 3.3% (95%-CI = 2.9–3.7) a subtype with strong related concerns. Report of memory-related SCS was unrelated to participants’ socio-demographic characteristics, physical comorbidity (except history of stroke), depressive symptomatology, and anxiety. Adults with and without memory-related SCS showed no significant difference in cognitive performance. About one fifth (18.1%) of the participants with memory-related SCS stated that they did consult/want to consult a physician because of their experienced memory problems.

**Conclusions:**

Memory-related SCS are very common and unspecific in the non-demented adult population aged 40–79 years. Nonetheless, a substantial proportion of this population has concerns related to experienced memory problems and/or seeks help. Already available information on additional features associated with a higher likelihood of developing dementia in people with SCS may help clinicians to decide who should be monitored more closely.

## Background

From a clinical point of view, a first step for early detection of a neurodegenerative process in primary care usually is to talk to the patient and ask whether he/she subjectively perceives problems in his/her cognitive function. General practitioners (GPs) or others may ask particularly for problems with memory as lay persons are rather familiar with this cognitive domain than with others (e.g., executive functioning, social cognition) and may also report problems with memory when they actually experience problems in another cognitive domain. In addition, it is necessary to ask for potentially memory-related concerns/worries as they have been found to be associated with an increased risk of progression to dementia [1]. It has been speculated that such concerns reflect a patient’s intuition that his or her subjective cognitive problems represent the beginning of a severe cognitive disorder rather than “normal aging” [2]. Second, the presence of such unpleasant or burdensome feelings would indicate to the GP and others to conduct a comprehensive examination of the experienced cognitive problems, which may include a detailed anamnesis regarding the subjectively perceived cognitive problems and a standardized cognitive screening/testing.

If objective cognitive deficits are observed, it should be investigated whether the patient may suffer from a milder cognitive syndrome like the well-established *Mild Cognitive Impairment* concept (MCI [3, 4]) or *Mild Neurocognitive Disorder* (NCD) according to criteria of the 5th edition of the *Diagnostic and Statistical Manual of Mental Disorders* (DSM-5; [5]) or even from a more severe cognitive syndrome like *Major NCD* according to DSM-5 criteria (a syndrome that incorporates the former DSM-IV diagnosis of dementia). Importantly, the diagnostic criteria of all these syndromes require the presence of subjectively perceived cognitive problems, amongst others.

If objective cognitive deficits are not present, it should be investigated, whether the patient suffers from a potential pre-prodromal syndrome in a neurodegenerative process. Research criteria for such a syndrome called *Subjective Cognitive Decline* (SCD) at the pre-prodromal stage in Alzheimer’s disease (AD) have been proposed recently by a *Subjective Cognitive Decline Initiative (SCD-I) Working Group* [6]. These research criteria, amongst others, require subjectively perceived cognitive problems (self-experienced persistent decline in cognitive capacity in comparison with a previously normal status and unrelated to an acute event). The SCD-I Working Group provided a list of core features for reporting in SCD studies and a list of features, which increase the likelihood of the presence of preclinical AD in individuals with SCD. Importantly, both lists contain the feature “concerns (worries) associated with SCD”.

### Study aims

Even though there is currently no cure for dementia, particularly of the most common AD type, an identification of subjectively perceived cognitive problems as a potential (pre-)prodrome may be important as it can enable people to plan ahead for the future, to make plans for care (e.g., power of attorney, advance directives for healthcare), or to make lifestyle changes that may slow the onset of cognitive decline. As a first step for early detection of a neurodegenerative process (i.e., to prove the presence/absence of a pre-prodromal (SCD) or prodromal stage (MCI/Mild NCD)) in primary care, as stated above, usually would be a question on subjectively perceived cognitive problems especially with regard to memory, the *overall aim* of this study was to provide detailed empirical information on such subjective cognitive problems in dementia-free adults. Following recently suggested terminologies [7], we will use the term *memory-related Subjective Cognitive Symptoms* (SCS) for the subjectively perceived memory problems that could be an indicator for a pre-prodromal (SCD) or prodromal stage (MCI/Mild NCD) of a neurodegenerative disease. Using data from a large German population-based sample, we sought

(i) to determine the prevalence of memory-related SCS in dementia-free adults aged 40–79 years (in total and for subtypes without, with some, and with strong concerns);
(ii) to analyze the association of the memory-related SCS with socio-demographic characteristics, physical comorbidity, objective cognitive performance, and depressive symptoms and anxiety (The analysis of the association with mood problems is particularly important as it has been shown that subjective memory complaints may be more related to mood problems than objective cognitive functioning. Individuals may experience a distorted subjective appraisal of their memory function in the presence of depressive symptoms. For more details, see [8]); and
(i) to provide further relevant information on memory-related SCS (e.g., areas of day-to-day life in which memory difficulties are experienced, onset of the symptoms, frequency of the experienced memory difficulties in day-to-day life, participants’ comparison of the own memory with the memory of adults of the same age, help seeking for memory difficulties).

## Methods

### Participants

Data were derived from the baseline (2011/08–2014/11) of the LIFE-Adult-Study. The LIFE-Adult-Study is a population-based cohort study investigating common chronic diseases and is conducted by the Leipzig Research Center for Civilization Diseases (LIFE) in Leipzig, Germany. The study design has been described in detail elsewhere [9, 10]. In brief, the study included an age- and sex-stratified random sample of *n* = 10,000 residents of the city of Leipzig covering an age range of 40–79 years (a subset of *n* = 400 participants age 18–39 years was also included). The residents were randomly selected from lists from the local registry office. Selected residents were sent an invitation letter including study information, a response form and a postage-paid return envelope. Residents who did not respond within 4 weeks received a reminder letter. Residents who did not respond to the second letter were searched in public telephone directories and contacted by phone. For residents who did not want to participate in the study, residents of the same age and sex were substitutionary randomly selected from the registry office’s lists and invited to participate. This procedure was repeated until the intended stratified sample size was reached. The final response rate was 33%.

### Data collection, assessment and classification procedures

All participants of the LIFE-Adult-Study underwent a comprehensive assessment program (day I). Participants aged ≥60 years were invited to participate in further assessments on additional days (day II-III) including, amongst others, a more extensive neuropsychological assessment [9]. In addition, an age-stratified subsample of 1200 participants aged 18–79 years were invited to participate in further assessments (e.g. abdominal MRI-scans, assessments of eating behavior) to investigate whether body fat distribution is associated with functional traits of the brain [9] and traits of eating behavior. In this particular subsample, some participants aged < 60 years were asked to take part in the extensive neuropsychological assessment usually conducted in participants aged ≥60 years (see also below, section on neuropsychological assessment). All assessments were conducted by trained study personnel. The entire assessment program was tested in a pilot study with approximately 400 participants. The assessments we analyzed data for the purpose of this report and the associated categorization procedures are described in the following sections.

#### Structured computer-assisted interview on socio-demographic and socio-economic information (day I)

The interview provided information on important socio-demographic characteristics of the participants such as age, sex, and education as well as (present, former) occupation and income. Based on the information on education, occupation and income, a socio-economic status (SES) index was calculated according to established criteria [11]. Based on the calculated SES-index, the participants were classified as having either a low, medium, or high SES.

#### Structured computer-assisted interviews on medical history (day I)

Participants were interviewed about medical diagnoses (> 70 common diseases) that were made by a physician [9]. For the purpose of this report, we included those diseases in analyses that may be relevant for memory-related SCS and cognitive performance: Parkinson’s disease, epilepsy, multiple sclerosis, cancer, diabetes mellitus, diseases of the thyroid gland, hyperlipidemia, hypertension, peripheral artery occlusive disease/intermittent claudicatio, cardiac arrhythmia, cardiac insufficiency, coronary heart disease/angina pectoris, myocardial infarction, and stroke.

#### Center of Epidemiologic Studies Depression Scale (CES-D; day I)

We identified depressive symptoms using this 20-item self-report screening instrument [12]. The maximum score of the CES-D is 60. Higher CES-D scores indicate more depressive symptoms. According to German reference values [13], a score of ≥23 indicates clinically relevant depressive symptomatology.

#### Generalized anxiety disorder screener (GAD-7; day I)

The GAD-7 is a 7-item self-report questionnaire to identify probable cases of generalized anxiety disorder and to assess the severity of anxiety symptoms [14, 15]. The maximum score of the GAD-7 is 21. Higher GAD-7 scores suggest more anxiety symptoms. A score of ≥10 indicates a probable generalized anxiety disorder.

#### Structured computer-assisted interview on memory-related SCS (day I)

Memory-related SCS was evaluated prior to cognitive testing. For this report, we analyzed data from the following questions: (i) “Do you feel as if your memory is becoming worse?” (No/Yes); (ii) “If yes, does this worry you?” (No/Yes, this does worry me/Yes, this does worry me very much). Based on participants’ response to question (i), participants were classified as having or not having memory-related SCS. Based on participants’ response to question (ii), participants were classified as having either memory-related SCS without concerns, with some concerns, or with strong concerns (memory-related SCS subtypes).

Further questions explored areas of daily living in which participants may have experienced memory-related SCS (No, not more difficult/Yes, a little bit more difficult/Yes, much more difficult): (iii) “Is it more difficult for you to remember recent events than in the past (when compared to 10 years ago)?”; (iv) “Is it more difficult for you to remember where you keep certain articles/things than in the past?”; (v) “Is it more difficult for you to remember the content of conversations that took place some days before than in the past?”; (vi) “Is it more difficult for you to remember appointments/dates than in the past?”; (vii) “Is it more difficult for you to remember names of acquaintances or friends than in the past?”

The following questions provided more detailed information on memory-related SCS: (viii) “Since when do you have the feeling your memory is becoming worse?” (Since less than 6 months/Since more than 6 months); (ix) “How often do the memory difficulties occur?” (Less than once a week/Once a week/Several times a week/Every day); (x) “Do you have the feeling that your memory is worse than the memory of adults of the same age?” (No/Yes); (xi) “Did you consult a physician in the past or do you plan to consult a physician in the future because of your memory difficulties?” (No/Yes).

#### Neuropsychological assessment

Neuropsychological assessment was conducted in the morning in a separate enclosed room. Instructions for the trained study personnel were computerized and participants’ test results were documented in an electronic data mask. The neuropsychological assessments were subject to regular quality control by experienced psychologists. On day I, all participants were first asked to answer the standardized questions on memory-related SCS and then to complete the following tests:

- *Verbal Fluency Test Animals* [16–19]: This test requires individuals to name as many animals as possible in 1 min and is used to measure verbal abilities and semantic memory.
- *Trail Making Test* (TMT; [20]): The TMT consists of two parts. TMT-A requires individuals to draw lines to connect consecutive numbers from 1 to 25 as fast as possible. TMT-B requires drawing lines to connect numbers and letters in an alternating sequence (1-A-2-B-3-C, etc.) as fast as possible. The time to complete each part is recorded. The time limit for participants to finish TMT-A is 180 s and to finish TMT-B 300 s. Shorter times indicate better cognitive performance. Performance on TMT-A is often used as a measure of attention or cognitive processing speed. Performance on TMT-B and the TMT ratio score B/A are used as measures of executive functioning.

Both Verbal Fluency Test Animals and the TMT are tests of the extended *Consortium to establish a registry for Alzheimer’s disease Neuropsychological Assessment Battery* (CERAD-NAB; [16, 17, 21]). The full battery was administered only to participants of the LIFE-Adult-Study aged ≥60 years and who took part in the extensive neuropsychological assessment on additional days [9] (see also above). However, some participants aged < 60 years of an aged-stratified subsample of 1200 participants were also asked to take part in a more extensive neuropsychological assessment – namely the CERAD-NAB tests *Word List Learning, Recall and Recognition*:

- *Word List Learning* (day II-III; [16, 17]): This test is used to assess the ability to learn and remember new verbal information. Individuals are asked to read aloud ten printed unrelated words presented at a rate of one every two seconds and then, immediately after presentation of the ten words to recall as many as possible. Two further trials are administered in this fashion with a different word order each trial. The maximum score of all trials together is 30.
- *Word List Recall* (day II-III; [16, 17]): This test is usually used to measure verbal memory/delayed free recall. It requires individuals to recall the ten words presented in the Word List Learning test. The maximum score is 10.
- *Word List Recognition* (day II-III; [16, 17, 22]): This test is usually used to measure verbal memory/delayed cued recall. The test requires individuals to recognize the ten words presented earlier in the Word List Learning test among ten other distractor words. The maximum score is 20 including the ten correctly recognized Word List Learning words and the ten correctly identified distractor words.

Altogether, we were able to conduct the three CERAD-NAB tests in *n* = 2756 (31.2%) participants aged 40–79 years (see below, Sample section). When analyzing the association between *memory*-related SCS and objective cognitive test performance, it is particular important to investigate the association with objective *memory* performance. Thus, we also included the results on the three CERAD-NAB memory tests in the analyses of this report even though we were only able to provide findings based on a subsample.

Participants who were unable to understand the test instructions (e.g., because of severe hearing impairment/deafness, insufficient German language skills, intellectual disability, possible dementia) were excluded from neuropsychological assessment. Depending on the underlying neuropsychological test, further exclusion criteria were applied, e.g., impaired vision, insufficient reading abilities (illiteracy etc.), and impaired motor function (because of Parkinson’s disease, paresis/stroke etc.) for the TMT, or communication/speech disorders (mutism, stuttering etc.) for the Verbal Fluency Test Animals. To exclude participants with dementia, we used the data from the extended CERAD-NAB to derive a possible diagnosis of Major NCD [5] (for details, see [10]).

### Statistical analysis

The statistical analyses were performed using IBM SPSS Statistics, version 24.0 (IBM Corp., Armonk, NY, USA). All analyses employed an alpha level for statistical significance of 0.05 (two-tailed).

First, we calculated point prevalence rates of memory-related SCS (total, age-, sex-, education-, SES-, and subtype-specific) as percentages with 95% confidence intervals (95%-CI). For calculation of the prevalence rates, we used weights which corrected for sample deviations in distribution from the adult population structure of Leipzig in 2012 with regard to age and sex (data provided by the Federal Statistical Office of Germany). To analyze the association of memory-related SCS with physical and mental comorbidity, we calculated the prevalence rates of memory-related SCS (total and subtypes) for adults with and without history of Parkinson’s disease, epilepsy, multiple sclerosis, cancer, diabetes mellitus, diseases of the thyroid gland, hyperlipidemia, hypertension, peripheral artery occlusive disease/intermittent claudicatio, cardiac arrhythmia, cardiac insufficiency, coronary heart disease/angina pectoris, myocardial infarction, stroke, current depressive symptomatology, and anxiety. Group differences were analyzed using the *χ*^*2*^ test. Moreover, univariate analysis of variance (ANOVA) and *t*-test were used to assess differences between adults with and without memory-related SCS and adults with the different memory-related SCS subtypes and adults without memory-related SCS with respect to depressive symptomatology and anxiety. In a supplementary analysis, we used multivariable logistic regression modelling to evaluate the association between having memory-related SCS and all covariates (socio-demographic characteristics, physical comorbidity, depressive symptomatology, anxiety, and cognitive performance; results are shown in Appendix 1).

Second, we provided more detailed information on memory-related SCS, namely on the onset of the SCS, the areas of daily living in which the SCS are experienced, the frequency of memory difficulties in day-to-day life, participants’ comparison of the own memory with the memory of adults of the same age, and the frequency of seeking help for memory-related SCS. Group differences were analyzed using the *χ*^*2*^*-* or *t-*test as appropriate.

Finally, we analyzed the association of memory-related SCS with cognitive performance in the Verbal Fluency Test Animals, Trail Making Test Part A (TMT-A; measuring attention or cognitive processing speed), TMT Part B (TMT-B) and TMT B/A (TMT-B and TMT B/A are measuring executive functioning. As stated above, in a subsample of *n* = 2756 (31.2%) participants aged 40–79 years, we were able to conduct the three CERAD-NAB tests Word List Learning, Recall and Recognition and we analyzed associations of memory-related SCS with performance in these three memory tests. ANOVA or *t*-test were used to assess potential differences in cognitive performance between (i) adults with and without memory-related SCS, (ii) adults with the different memory-related SCS subtypes and adults without memory-related SCS, (iii) adults with memory-related SCS with different frequency of memory difficulties in day-to-day life, (iv) adults with memory-related SCS who sought/seek help for memory-related SCS and adults with memory-related SCS who didn’t/don’t seek, and (v) adults with memory-related SCS who rated their memory as being worse than the memory of adults of the same age and adults with memory-related SCS who rated their memory as being not worse than the memory of others.

If necessary, the Bonferroni correction procedure for adjustments for multiple testing was applied.

## Results

### Sample

Among the LIFE-Adult-Study sample of *n* = 10,000 adults, we excluded the *n* = 400 participants from analyses who participated in the pilot study. Among the remaining participants, we excluded participants who were younger than 40 years and for which no information on memory-related SCS or sociodemographic characteristics could be obtained, leaving a final analysis pool of *n* = 8834 adults: *n* = 4607 (52.2%) women and *n* = 4227 men (47.8%). The mean age of the sample was 58.8 years (SD = 11.0). One third (*n* = 2966/33.6%) of the participants had a university degree. The majority of the participants (*n* = 5317/60.2%) had a medium SES (low SES: *n* = 1752/19.8%; high SES: *n* = 1765/20.0%). Participants of the study sample and participants who had to be excluded because of missing information on memory related SCS did not differ in sex (*χ*^2^ = 1.585, df = 1, *p* = 0.208), age (*t* = 0.837, *p* = 0.403), education (*χ*^2^ = 0.049, df = 1, *p* = 0.825), or SES (*χ*^2^ = 2.606, df = 2, *p* = 0.272).

### Prevalence of memory-related SCS and association with socio-demographic characteristics

Overall, *n* = 4678 (53.0%) of the *n* = 8834 participants stated to have memory-related SCS. Regarding subtypes, *n* = 2296 (26.0%) had memory-related SCS without concerns, *n* = 2087 (23.6%) with some concerns, and *n* = 295 (3.3%) with strong concerns. Analysis corrected for age- and sex-related sample deviations from the adult population structure of Leipzig resulted in corresponding weighted prevalence rates of 53.0% for total memory-related SCS (95%-CI = 51.9–54.0), 26.0% (95%-CI = 25.1–27.0) for memory-related SCS without concerns, 23.6% (95%-CI = 22.7–24.5) for memory-related SCS with some concerns, and 3.3% (95%-CI = 2.9–3.7) for memory-related SCS with strong concerns. Prevalence rates of total memory-related SCS did not differ significantly with regard to age, sex, education, or SES of the adult population (Table 1; see also Appendix 1). The same was found for the prevalence rates of the memory-related SCS subtypes (results of overall *χ*^*2*^ tests on all *n* = 8834 participants for SCS subtype prevalence differences regarding age group: *χ*^2^ = 4.270, df = 9, *p* = 0.893; sex: *χ*^2^ = 3.556, df = 3, *p* = 0.314; education: *χ*^2^ = 3.855, df = 3, *p* = 0.278; SES: *χ*^2^ = 6.334, df = 6, *p* = 0.387; age, sex-, education-, and SES-specific prevalence rates of the subtypes are therefore not presented).

**Table 1:** Prevalence rates of memory-related SCS (*n* = 8834)

	Prevalence^a^		df	*χ* ^2^	*p* value
	%	95%-CI			
Memory-related SCS total	53.0	51.9–54.0			
Men	53.4	51.9–54.9	1	0.577	0.448
Women	52.6	51.1–54.0			
Age group 40–49 years	54.3	52.4–56.2	3	3.722	0.293
Age group 50–59 years	51.7	49.7–53.7			
Age group 60–69 years	52.3	50.1–54.6			
Age group 70–79 years	53.3	51.1–55.4			
Without university degree	52.9	51.7–54.2	1	0.016	0.900
With university degree	53.1	51.2–54.9			
Low SES	52.1	49.7–54.4	2	3.369	0.186
Medium SES	53.7	52.4–55.1			
High SES	51.5	49.2–53.8			
Memory-related SCS without concerns	26.0	25.1–27.0			
Memory-related SCS with some concerns	23.6	22.7–24.5			
Memory-related SCS with strong concerns	3.3	2.9–3.7			

^a^For calculation of the prevalence rates, weights were used which corrected for sample deviations in distribution from the adult population structure of the city of Leipzig in 2012 with regard to age and sex

*CI* confidence interval, *df* degree of freedom, *SES* socio-economic status, *SCS* subjective cognitive symptoms

### Association of memory-related SCS with physical comorbidity

Information on the selected physical comorbidities was collected for *n* = 8036 (91.0%) of the n = 8834 participants in the study sample. Among those, 44.8% (95%-CI = 43.7–45.9) reported to have a history of arterial hypertension, 34.2% (95%-CI = 33.1–35.2) hyperlipidemia, 28.3% (95%-CI = 27.4–29.3) thyroid disease, 10.9% (95%-CI = 10.2–11.5) cardiac arrhythmia, 10.5% (95%-CI = 9.9–11.2) diabetes mellitus, 10.5% (95%-CI = 9.8–11.2) any cancer, 3.1% (95%-CI = 2.7–3.5) coronary heart disease/angina pectoris, 2.2% (95%-CI = 1.9–2.5) myocardial infarction, 2.1% (95%-CI = 1.8–2.4) stroke, 2.0% (95%-CI = 1.7–2.3) cardiac insufficiency, 1.5% (95%-CI = 1.2–1.7) epilepsy, 1.0% (95%-CI = 0.8–1.2) peripheral artery occlusive disease/intermittent claudication, 0.3% (95%-CI = 0.2–0.4) Parkinson’s disease, and 0.3% (95%-CI = 0.2–0.5) multiple sclerosis. For all of these diseases, we did not find any significant difference in the weighted prevalence of memory-related SCS (results are presented in Appendix 2; see also Appendix 1). We also found no significant difference in the weighted prevalence rates of memory-related SCS subtypes between adults with the disease and adults without, with one exception (results are presented in Appendix 3): adults with a history of stroke had a lower prevalence of memory-related SCS without concerns than adults without a stroke (16.3%/95%-CI = 10.7–21.9 vs. 26.5%/95%-CI = 25.5–27.5) but higher prevalence rates of memory-related SCS with some concerns (29.5%/95%-CI = 22.6–36.5 vs. 23.8%/95%-CI = 22.8–24.7) and strong concerns (5.4%/95%-CI = 2.0–8.9 vs. 3.3%/95%-CI = 2.9–3.6). The overall *χ*^2^ test for comparing these six prevalence rates yielded a *χ*^2^ = 11.185 (df = 3, *p* = 0.011; *n* = 8034).

### Association of memory-related SCS with depressive symptoms and anxiety

Information on depressive symptoms (CES-D) was collected for *n* = 7854 (88.9%) of the *n* = 8834 participants in the study sample. The mean CES-D score was 10.8 (SD = 6.9). Participants with and without memory-related SCS did not differ significantly in the CES-D score (mean/SD = 10.9/7.0 vs. 10.7/6.8; *t* = − 0.871, *p* = 0.384). Moreover, participants with different memory-related SCS subtypes and participants without memory-related SCS did not differ significantly with respect to their CES-D score (memory-related SCS without concerns: mean/SD CES-D score = 10.7/6.8; memory-related SCS with some concerns: 11.0/7.0; memory-related SCS with strong concerns: 11.4/7.6; *F* = 1.248, *p* = 0.290).

The weighted prevalence of depressive symptomatology according to the previously validated German CES-D cut-off score of ≥23 points was 6.3% (95%-CI = 5.8–6.8). Adults with depressive symptomatology showed no significantly higher weighted prevalence of memory-related SCS than adults without (57.2%/95%-CI = 52.8–61.6 vs. 52.7%/95%-CI = 51.5–53.8; *χ*^*2*^ = 3.785, df = 1, *p* = 0.052, *n* = 7854; see also Appendix 1). Regarding subtypes, in adults with depressive symptomatology, the weighted prevalence rates were 26.2% (95%-CI = 22.3–30.0) for memory-related SCS without concerns, 26.8% (95%-CI = 22.9–30.7) for memory-related SCS with some concerns and 4.3% (95%-CI = 2.5–6.0) for memory-related SCS with strong concerns, respectively. In adults without depressive symptomatology, the corresponding weighted prevalence rates were 26.1% (95%-CI = 25.1–27.1), 23.4% (95%-CI = 22.4–24.4) and 3.2% (95%-CI = 2.8–3.6), but the differences in the rates were also not statistically significant (results of overall *χ*^*2*^ test for comparing these six prevalence rates: *χ*^*2*^ test: *χ*^*2*^ = 5.848, df = 3, *p* = 0.119, n = 7854).

Information on anxiety (GAD-7) was collected for *n* = 8476 (95.9%) of the *n* = 8834 participants in the study sample. The weighted prevalence of possible generalized anxiety disorder according to the GAD-7 cut-off score of ≥10 points was 5.9% (95%-CI = 5.4–6.4).

The mean GAD-7 score was 3.5 (SD = 3.4). Participants with and without memory-related SCS did not differ significantly in the GAD-7 score (mean/SD = 3.6/3.4 vs. 3.5/3.4; *t* = − 1.394, *p* = 0.163). Participants with different memory-related SCS subtypes and participants without memory-related SCS did not differ significantly in the GAD-7 score (memory-related SCS without concerns: mean/SD GAD-7 score = 3.6/3.4; memory-related SCS with some concerns: 3.5/3.4; memory-related SCS with strong concerns: 3.6/3.3; *F* = 0.932, *p* = 0.424).

The weighted prevalence of memory-related SCS, in adults with possible generalized anxiety disorder, was 55.7% (95%-CI = 51.4–60.1) and, in adults without, 52.8% (95%-CI = 51.7–53.9); the difference was not statistically significant (*χ*^*2*^ = 1.611, df = 1, *p* = 0.204, *n* = 8476; see also Appendix 1). There were also no statistically significant differences with respect to the SCS subtypes: In adults with possible generalized anxiety disorder, the prevalence of memory-related SCS without concerns was 27.1% (95%-CI = 23.2–31.0), the prevalence of memory-related SCS with some concerns 24.7% (95%-CI = 20.9–28.5), and the prevalence of memory-related SCS with strong concerns 3.8% (95%-CI = 2.1–5.5). In adults without possible generalized anxiety disorder the corresponding prevalence rates were 25.9% (95%-CI = 25.0–26.9), 23.6% (95%-CI = 22.7–24.6), and 3.2% (95%-CI = 2.9–3.6). The overall *χ*^*2*^ test for comparing these six prevalence rates yielded *a χ*^*2*^ = 1.752 (df = 3, *p* = 0.626, *n* = 8476).

### Areas of memory-related SCS

We asked participants who reported memory-related SCS about the events of daily living during which they experienced the memory difficulties. A decline in memory was most frequently experienced with respect to remembering recent events (58.6% with some more or much more difficulties than in the past) and remembering names of acquaintances or friends (56.1%; Table 2). By contrast, a memory decline was less frequently experienced with respect to remembering appointment/dates (29.6% with some more or much more difficulties than in the past).

**Table 2:** Areas of memory-related SCS (*n* = 4679)^a^

“Is it more difficult for you to remember…”	No, not more difficult (%)	Yes, a little bit more difficult (%)	Yes, much more difficult (%)	I don’t know (%)
“… appointments/dates than in the past^b^?”	69.9	26.7	2.9	0.5
“… where you keep certain articles/things than in the past?”	49.6	45.2	5.0	0.3
“… the content of conversations that took place some days before than in the past?”	48.9	44.9	5.3	0.9
“… names of acquaintances or friends than in the past?”	43.6	46.6	9.5	0.3
“… recent events than in the past?”	40.8	53.4	5.2	0.6

^a^Weights were used which corrected for sample deviations in distribution from the adult population structure of the city of Leipzig in 2012 with regard to age and sex. Weighting resulted in a sample size of n = 4679 instead of 4678 participants with memory-related SCS

^b^’past’ was always specified as ‘when compared to 10 years ago’

*SCS* subjective cognitive symptoms

### Onset of memory-related SCS and further information on the experienced memory difficulties

The majority (87.4%) of the participants with memory-related SCS stated that they are experiencing memory difficulties since more than six months (10.7% since less than six months; 1.9% didn’t know/no answer). Half (50.4%) of the participants with memory-related SCS stated to have difficulties with memory less than once a week, 24.2% once a week, 13.5% several times a week, and 7.2% every day (4.7% didn’t know/no answer).

When asking the participants whether they think that their own memory is worse than the memory of adults of the same age, 9.5% answered the question with yes (85.7% with no; 4.8% didn’t know/no answer). Those participants who rated their memory as being worse than the memory of others had significantly more often memory-related SCS with some concerns or with strong concerns than those who rated their memory as being not worse than the memory of others (60.2%/24.9% vs. 42.4%/3.4%; *χ*^2^ = 477.399, df = 2, *p* < 0.001). There were no significant differences between these two groups with regard to age (*t* = 1.743, *p* = 0.081), sex (*χ*^*2*^ = 0.136, df = 1, *p* = 0.712), education (*χ*^*2*^ = 0.233, df = 1, *p* = 0.630), or SES (*χ*^*2*^ = 1.346, df = 2, *p* = 0.510).

### Seeking help for memory-related SCS

About one fifth (18.1%) of the participants with memory-related SCS stated that they did consult or want to consult a physician because of their memory difficulties (81.6% no consultation in the past/in the future; 0.3% didn’t know/no answer). Those participants who consulted or wanted to consult a physician had significantly more often memory-related SCS with some concerns or with strong concerns than those who didn’t consult/don’t want to consult (60.5%/19.7% vs. 41.0%/3.2%; *χ*^*2*^ = 542.462, df = 2, *p* < 0.001). There were no significant differences between these two groups with regard to age (*t* = − 0.756, *p* = 0.450), sex (*χ*^*2*^ = 0.037, df = 1, *p* = 0.847), education (*χ*^*2*^ = 0.757, df = 1, *p* = 0.384), or SES (*χ*^*2*^ = 3.895, df = 2, *p* = 0.143).

### Association of memory-related SCS with cognitive performance

Reliable test results on the Verbal Fluency Test Animals were obtained for *n* = 8803 (99.6%) of the *n* = 8834 participants. The mean number of animals named in one minute was 23.7 (SD = 6.5). Reliable test results on the TMT-A and B were obtained for *n* = 8594 (97.3%) participants. The mean time needed to complete the TMT-A was 37.3 s (SD = 15.3) and to complete the TMT-B 90.2 s (SD = 47.3). The mean ratio score TMT B/A in the study sample was 2.5 (SD = 1.0). Participants with and without memory-related SCS showed comparable performance in the Verbal Fluency Test, TMT-A, TMT-B, and TMT B/A (Table 3; see also Appendix 1). Likewise, no differences in cognitive performance were found with regard to memory-related SCS subtypes.

**Table 3:** Association of memory-related SCS with cognitive performance^a^ (*n* = 8834)

	Total sample	Memory-related SCS total		Memory-related SCS subtypes			
		Yes	No	Without concerns	With some concerns	With strong concerns	No memory-related SCS
Verbal Fluency Test Animals^b^							
No. of animals	23.7 (6.5)	23.7 (6.4)	23.7 (6.5)	23.8 (6.4)	23.5 (6.5)	24.0 (6.5)	23.7 (6.5)
*t; F*		−0.064		0.997			
*p* value		0.949		0.393			
Trail Making Test A^c^							
Sec, mean (SD)	37.3 (15.3)	37.1 (15.4)	37.5 (15.5)	36.9 (14.9)	37.5 (16.1)	36.5 (14.2)	37.5 (15.5)
*t; F*		1.012		1.085			
*p* value		0.312		0.354			
Trail Making Test B^c^							
Sec, mean (SD)	90.2 (47.3)	90.0 (47.6)	91.0 (47.7)	88.5 (45.4)	91.5 (49.6)	91.1 (49.7)	91.0 (47.7)
*t; F*		0.914		1.675			
*p* value		0.361		0.170			
Trail Making Test B/A^c^							
Ratio score	2.5 (1.0)	2.5 (1.0)	2.5 (1.0)	2.5 (0.9)	2.5 (1.0)	2.5 (1.0)	2.5 (1.0)
*t; F*		0.348		0.624			
*p* value		0.805		0.599			
Word List Learning^d^							
No. of words	21.8 (3.8)	21.9 (3.8)	21.7 (3.8)	22.0 (3.8)	21.8 (3.9)	22.0 (3.5)	21.7 (3.8)
*t; F*		−1.220		0.702			
*p* value		0.223		0.551			
Word List Recall^d^							
No. of words	7.7 (1.8)	7.7 (1.9)	7.7 (1.7)	7.7 (1.8)	7.7 (1.9)	7.3 (2.0)	7.7 (1.7)
*t; F*		0.742		1.584			
*p* value		0.458		0.191			
Word List Recognition^d^							
No. of words	19.7 (0.8)	19.6 (0.8)	19.7 (0.8)	19.6 (0.9)	19.7 (0.7)	19.5 (1.3)	19.7 (0.8)
*t; F*		0.913		0.845			
*p* value		0.361		0.469			

^a^Weighting factors were used which corrected for sample deviations from the adult population structure of the city of Leipzig in 2012 with regard to age and sex

^b^missing data for *n* = 31 (0.3%) of the 8834 participants

^c^missing data for *n* = 240 (2.7%) of the n = 8834 participants

^d^The CERAD-NAB memory tests Word List Learning, Recall and Recognition were conducted in a subsample of n = 2756 (31.2%) participants

*No*. number, *Sec*. seconds, *SCS* subjective cognitive symptoms

As stated in the methods’ section, a subsample of *n* = 2756 (31.2%) participants additionally conducted the three memory-related Word List tests of the extended CERAD-NAB. There were no significant differences in the results of these tests between participants with and without memory-related SCS or with regard to memory-related SCS subtypes (Table 3).

In three sub-analyses of participants with memory-related SCS, we compared the performances in the cognitive tests of:

(i) participants who reported to have difficulties with memory less than once a week, once a week, several times a week, and every day,
(ii) participants who rated their memory as being worse than the memory of adults of the same age and participants who rated their memory as not being worse, and
(iii) participants who consulted or want to consult a physician and participants who didn’t consult/don’t want to consult a physician.

We did not find any significant difference in cognitive performance between the groups except a slight, but significantly higher mean TMT B/A (indicating worse test performance) in the group of participants with memory-related SCS who rated their own memory being worse compared to those who did not (mean/SD = 2.6/1.1 vs. 2.5/0.9; *t* = − 2.030, *p* = 0.043; the non-significant results for the other cognitive test results are not shown).

## Discussion

In this study, we sought to provide detailed empirical information on *memory-related Subjective Cognitive Symptoms* (SCS) in the general adult population.

Using data from a large German population-based sample of dementia-free adults aged 40–79 years, we followed three aims. Our first aim was to provide information on the occurrence of memory-related SCS in this population group. We found a total prevalence of 53.0%. This rate corroborates findings from two previous large community-based studies indicating a high prevalence of memory-related SCS in the general adult population. Holmen et al. [23] observed rates of 44.6%/46.2% for subjectively perceived minor memory problems in a Norwegian sample of women/men, and Singh-Manoux et al. [24] reported a prevalence of 56.3% for subjectively perceived memory problems in a French sample. Lower rates have been reported by others (e.g., 21.9–31.7%; [25–27]). Begum et al. [28] observed prevalence rates of 7.4, 8.7, and 9.4% for subjective memory complaints in English surveys conducted in 1993, 2000, and 2007 respectively. However, a review has shown that prevalence rates of memory complaints can vary largely between studies (25–50%) [29]. One of the most important factors that contributes to this variation is the large variety of definitions and assessment strategies for subjective cognitive/memory complaints/deficits/decline/ impairment [1, 30, 31]. The above cited studies are comparable to each other because they usually did not exclude participants with objective cognitive deficits/MCI (in two studies, participants with dementia were excluded [26, 27]). Nonetheless, there are substantial differences: Regarding the low prevalence for subjective memory complaints in the study of Begum et al. [28], for example, it is important to know that these rates referred only to subjectively perceived memory problems noticed at least one day in the preceding week, whereas in our study and others [23, 24] no such time criterion was applied. A comparison of the prevalence rates therefore has to be made with caution.

Regarding the prevalence of different subtypes of memory-related SCS, we found that in about half of the adults memory-related SCS were accompanied by concern. As stated above, such concerns or worries can be important as they may reflect a patient’s intuition that his or her subjective cognitive problems represent the beginning of a severe cognitive disorder rather than “normal aging” [2]. Further, such concerns have been found to be associated with a significantly increased risk of progression to dementia compared to memory-related SCS/SCD without concerns/worries (for an overview, see [1]; for details, see [2, 32–34]). Concerns are unpleasant/burdensome feelings, and may thus indicate to GPs and others to conduct a comprehensive examination of the SCS. However, it is important to note that concerns or worries about SCS can also be an artifact of mood problems. As stated above, it has been shown that subjective memory complaints may be more related to mood problems like anxiety or depressive symptoms than to objective cognitive function (for more details, see [8]).

Our second aim was to provide additional information on factors associated with memory-related SCS: Importantly, memory-related SCS (total as well as subtypes) was completely unrelated to participants’ socio-demographic characteristics or physical comorbidity (except history of stroke), depressive symptomatology, or anxiety. Moreover, there was no significant difference in cognitive performance between adults with and without memory-related SCS (or with respect to subtypes). Regarding associations with socio-demographic characteristics, previous studies do not provide a clear picture. Older age, for example, was found to be associated with a higher risk/higher prevalence in several studies (e.g. [23, 26]), but not in all [27]. Regarding sex, some studies have reported higher rates of subjectively perceived memory problems/decline for women than men (e.g. [24, 26]), whereas others reported higher rates for men than women (e.g. [23]) or comparable rates (e.g. [27]). With regard to education, one study observed higher rates for people with lower education [23], whereas others observed more complex [25] or no associations [27]. Findings on associations of subjectively perceived memory problems with objective cognitive performance have been somewhat inconsistent, but several studies, including cross-sectional ones, observed significant associations [26, 35]. More consistent findings have been shown by previous studies regarding associations between subjectively perceived memory problems/decline and symptoms of depression (e.g. [23, 24, 27, 36] and anxiety (e.g. [23, 27]). As we observed none of such associations and also no association with physical comorbidity (except history of stroke), our findings provide further evidence that a general report of memory-related SCS (at least at the population level) is a rather unspecific phenomenon in adults aged 40–79 years.

Looking at memory-related SCS in more detail (third study aim), we found that (i) 44.9% of the adults with memory-related SCS stated to have difficulties with memory at least once a week, (ii) 9.5% rated their memory as being worse than the memory of adults of the same age, and (iii) 18.1% stated that they did consult or want to consult a physician because of their memory difficulties. We then compared cognitive performances in adults with memory-related SCS who (i) reported to have difficulties with memory less frequently, (ii) rated their memory as being not worse than the memory of adults of the same age, and (iii) who didn’t consult/don’t want to consult a physician because of their experienced memory-related SCS. But we did not find any significant difference. However, it is important to note that we were only able to provide cross-sectional findings, and cannot derive a meaning for clinical interest. Findings from longitudinal prospective studies may be more suitable, especially as ‘subjective feelings of worse performance than others of the same age group’ have been found to increase the likelihood of preclinical AD [6].

We wish to acknowledge further limitations: First, only a fraction of the people invited to the LIFE-Adult-Study responded, and some participants had to be excluded from analyses because of incomplete assessment information. Even though we aimed to correct for sample deviations by using weights, a possible selection bias cannot completely be excluded. It is, for example, possible that individuals who did not participate in the study had either more or less often memory-related SCS and/or were either cognitively more or less impaired than those who did. However, as recently published findings of the LIFE-Adult-Study [10] showed a prevalence of MCI [3, 4] that strongly conforms with the average prevalence in major population-based studies, we assume that there is not a strong selection bias of our results. Second, we are also fully aware that the provided weighted prevalence rates of memory-related SCS and the respective associations are dependent on the methods used to assess SCS. Comparison of our results with results of other studies has to take this into account.

## Conclusions

Irrespective of these limitations, we think that our findings derived from a large population-based sample of non-demented adults aged 40–79 years are robust enough to support the notion that memory-related SCS are very common and unspecific in dementia-free adults of the general population. However, a substantial proportion of this population has concerns about their memory-related SCS and/or seeks help. This has *clinical-practical implications*: Regardless of the unspecific character of memory-related SCS, clinicians have to pay attention to such subjective symptoms. Comprehensive examinations of the SCS may be required to collect information on whether the concerns about SCS are just an artifact of mood problems [8] and whether additional features known to be associated with a higher likelihood of developing dementia are present. As shown by Jessen et al. [6] for the SCD-syndrome as a potential pre-prodromal stage in AD, the likelihood of preclinical AD in individuals with SCD is increased by the following features: SCD onset within the last 5 years, age at onset ≥60 years, confirmation of cognitive decline by an informant, presence of the APOE ε4 genotype, and biomarker evidence for AD. Such information may help clinicians at least to decide who should be monitored more closely. From a theoretical point of view (*theoretical implications*), the high prevalence and the unspecific character of general memory-related SCS emphasize the necessity of identifying and defining SCS more specifically for certain clinical outcomes (e.g., SCD in preclinical AD [6]) and to separate the SCS subtypes from each other (e.g. SCD in preclinical AD vs. SCS due to mood problems).

## Appendix 1

**Table 4:** Multivariable logistic regression on the association between having memory-related SCS and covariates (n=6,960)^1;2^

Covariates^3^	df	Wald’s *χ*²	*p* value	OR	95%-CI
Sex, women vs. men	1	2.686	0.101	0.92	0.83-1.03
Age, every additional year	1	0.705	0.401	1.00	0.99-1.00
Education, with university degree vs. without	1	1.901	0.168	1.10	0.96-1.26
SES					
Medium vs. low	1	0.115	0.735	1.02	0.90-1.17
High vs. los	1	2.727	0.099	0.84	0.69-1.03
Physical comorbidity, yes vs. no					
Arterial hypertension	1	0.163	0.687	0.98	0.88-1.09
Hyperlipidemia	1	0.443	0.506	0.96	0.87-1.07
Thyroid disease	1	2.960	0.085	1.10	0.99-1.24
Cardiac arrhythmia	1	1.044	0.307	1.09	0.93-1.28
Diabetes mellitus	1	0.165	0.684	1.04	0.88-1.23
Any cancer	1	0.576	0.448	0.94	0.80-1.11
Coronary heart disease/angina pectoris	1	0.017	0.897	1.02	0.75-1.40
Myocardial infarction	1	1.723	0.189	1.28	0.89-1.84
Stroke	1	0.001	0.972	1.01	0.70-1.44
Cardiac insufficiency	1	0.032	0.857	0.97	0.68-1.38
Epilepsy	1	1.620	0.203	0.77	0.51-1.15
Peripheral artery occlusive disease/intermittent claudication	1	0.495	0.482	0.84	0.51-1.37
Parkinson’s disease	1	0.312	0.576	0.76	0.29-1.98
Multiple sclerosis	1	0.311	0.577	0.79	0.35-1.81
Depressive symptomatology					
CES-D score, every additional point	1	0.054	0.816	1.00	0.99-1.01
Anxiety					
GAD-7 score, every additional point	1	0.446	0.504	1.01	0.99-1.03
Cognition^3^					
VFT, every additional stated animal	1	0.026	9.871	1.00	0.99.1.01

^1^missing data for n = 1,874 (21.2%) of the 8,834 participants; ^2^Nagelkerke’s R² of the model = 0.004; ^3^The cognitive test results of Trail Making Test (TMT) A, TMT B and TMT ratio score B/A could not be additionally included into this regression model (model I) because the cognitive test results were not independent from each other. As a result, three additional regressions models (II-IV) were calculated including TMT A result (model II), TMT B result (model III), and TMT ratio score B/A result (model IV) instead of VFT test result as cognitive covariate. In these models, the different TMT tests results were also not significantly associated with having memory-related SCS: Every additional second in TMT A yielded an OR = 1.00 (95%-CI = 0.99-1.00; Wald’s *χ*² = 0.486, df=1, p=0.486; model II), every additional second in TMT B yielded an OR = 1.00 (95%-CI = 1.00-1.00; Wald’s *χ*² = 0.100, df=1, p=0.752; model III), and higher TMT ratio score B/A an OR =1.00 (95%-CI = 0.95-1.05; Wald’s *χ*² = 0.010, df=1, p=0.922; model IV). The replacement of the VFT test score by the other cognitive test results in regression model II-IV did not change the (non-significant) association of the other covariates with having memory-related SCS

*CES-D* Center of Epidemiologic Studies Depression Scale, *CI* confidence interval, *GAD-7* Generalized Anxiety Disorder Screener, *df* degree of freedom, *OR* odds ratio, *SES* socio-economic status, *SCS* subjective cognitive symptoms *VFT* Verbal Fluency Test

## Appendix 2

**Table 5:** Prevalence rates of memory-related SCS in relation to the presence/ absence of physical comorbidity (n=8,036)

Physical comorbidity		Prevalence of memory-related SCS^1^ (%)	*χ*²	df	*p* value
Arterial hypertension	Yes	53.0	0.571	1	0.450
	No	53.9			
Hyperlipidemia	Yes	53.2	0.118	1	0.731
	No	53.6			
Thyroid disease	Yes	55.1	3.363	1	0.067
	No	52.8			
Cardiac arrhythmia	Yes	55.0	0.897	1	0.344
	No	53.3			
Diabetes mellitus	Yes	52.9	0.128	1	0.720
	No	53.5			
Any cancer	Yes	52.7	0.245	1	0.621
	No	53.6			
Coronary heart disease/angina pectoris	Yes	55.8	0.573	1	0.449
	No	53.4			
Myocardial infarction	Yes	59.6	2.702	1	0.100
	No	53.3			
Stroke	Yes	51.2	0.351	1	0.554
	No	53.5			
Cardiac insufficiency	Yes	53.5	0.000	1	0.997
	No	53.5			
Epilepsy	Yes	47.9	1.502	1	0.220
	No	53.6			
Peripheral artery occlusive disease/ intermittent claudication	Yes	50.6	0.258	1	0.612
	No	53.5			
Parkinson’s disease	Yes	54.2	0.005	1	0.946
	No	53.5			
Multiple sclerosis	Yes	51.9	0.029	1	0.866
	No	53.5			

^1^For calculation of the prevalence rates, weights were used which corrected for sample deviations in distribution from the adult population structure of the city of Leipzig in 2012 with regard to age and sex

*df* degree of freedom, *SCS* subjective cognitive symptoms

## Appendix 3

**Table 6:** Prevalence rates of memory-related SCS subtypes in relation to the presence/absence of physical comorbidity (n=8,036)

Physical comorbidity		Prevalence of mr SCS without concerns^1^ (%)	Prevalence of mr SCS with some concerncs^1^ (%)	Prevalence of mr SCS with strong concerncs^1^ (%)	*χ*²	df	*p* value
Arterial hypertension	Yes	26.3	23.5	3.2	0.876	3	0.831
	No	26.2	42.2	3.4			
Hyperlipidemia	Yes	25.9	23.4	3.9	5.076	3	0.166
	No	26.4	24.2	3.0			
Thyroid disease	Yes	27.2	24.9	3.0	5.127	3	0.163
	No	25.9	23.5	3.4			
Cardiac arrhythmia	Yes	27.2	24.8	3.0	1.546	3	0.672
	No	26.2	23.8	3.4			
Diabetes mellitus	Yes	25.7	24.6	2.6	1.817	3	0.611
	No	26.3	23.8	3.4			
Any cancer	Yes	24.6	24.2	3.9	2.271	3	0.518
	No	26.5	23.9	3.2			
Coronary heart disease/angina pectoris	Yes	30.4	22.4	3.2	2.273	3	0.518
	No	26.1	23.9	3.3			
Myocardial infarction	Yes	27.5	27.5	4.5	3.329	3	0.344
	No	26.3	23.8	3.3			
Stroke	Yes	16.3	29.5	5.4	11.185	3	0.011
	No	26.5	23.8	3.3			
Cardiac insufficiency	Yes	30.2	22.0	1.3	3.249	3	0.355
	No	26.2	23.9	3.4			
Epilepsy	Yes	19.7	26.5	1.7	4.040	3	0.257
	No	26.4	23.8	3.3			
Peripheral artery occlusive disease/ intermittent claudication	Yes	28.8	21.3	1.3	1.546	3	0.672
	No	26.3	23.9	3.3			
Parkinson’s disease	Yes	20.8	29.2	4.2	0.608	3	0.895
	No	26.3	23.9	3.3			
Multiple sclerosis	Yes	29.6	18.5	3.7	0.469	3	0.926
	No	26.3	23.9	3.3			

^1^For calculation of the prevalence rates, weights were used which corrected for sample deviations in distribution from the adult population structure of the city of Leipzig in 2012 with regard to age and sex

*df* degree of freedom, *mr* memory-related, *SCS* subjective cognitive symptoms

## Abbreviations

AD: Alzheimer’s disease
CERAD-NAB: Consortium to establish a registry for Alzheimer’s disease Neuropsychological assessment battery
CES-D: Center of epidemiologic studies depression scale
CI: Confidence interval
DSM: Diagnostic and statistical manual of mental disorders
GAD-7: Generalized anxiety disorder screener
GP: General Practitioners
MCI: Mild cognitive impairment
NCD: Neurocognitive disorder
SCD-I: Subjective cognitive decline initiative
SCS: Subjective cognitive symptoms
SD: Standard deviation
SES: Socio-economic status
TMT: Trail making test
